# Up-regulation of *miR-98* and unraveling regulatory mechanisms in gestational diabetes mellitus

**DOI:** 10.1038/srep32268

**Published:** 2016-08-30

**Authors:** Jing-Li Cao, Lu Zhang, Jian Li, Shi Tian, Xiao-Dan Lv, Xue-Qin Wang, Xing Su, Ying Li, Yi Hu, Xu Ma, Hong-Fei Xia

**Affiliations:** 1 Reproductive and Genetic Center of National Research Institute for Family Planning, Beijing, 100081, China; 2 Graduate School, Peking Union Medical College, Beijing, 100730, China; 3 Third Affiliated Hospital of Beijing University of Chinese Medicine, Beijing, 100029, China; 4 Maternal and Child Health Hospital, Haidian District, Beijing, 100080, China

## Abstract

*MiR-98* expression was up-regulated in kidney in response to early diabetic nephropathy in mouse and down-regulated in muscle in type 2 diabetes in human. However, the expression prolife and functional role of *miR-98* in human gestational diabetes mellitus (GDM) remained unclear. Here, we investigated its expression and function in placental tissues from GDM patients and the possible molecular mechanisms. The results showed that *miR-98* was up-regulated in placentas from GDM patients compared with normal placentas. *MiR-98* over-expression increased global DNA methylational level and *miR-98* knockdown reduced global DNA methylational level. Further investigation revealed that *miR-98* could inhibit *Mecp2* expression by binding the 3′-untranslated region (UTR) of methyl CpG binding protein 2 (*Mecp2*), and then led to the expression dysregulation of canonical transient receptor potential 3 (*Trpc3*), a glucose uptake related gene. More importantly, *in vivo* analysis found that the expression level of *Mecp2* and *Trpc3* in placental tissues from GDM patients, relative to the increase of *miR-98*, was diminished, especially for GDM patients over the age of 35 years. Collectively, up-regulation of *miR-98* in the placental tissues of human GDM is linked to the global DNA methylation *via* targeting *Mecp2,* which may imply a novel regulatory mechanism in GDM.

Gestational diabetes mellitus (GDM) is a condition in which women without previously diagnosed diabetes exhibit varying degrees of glucose intolerance during pregnancy^1^. In different studies, gestational diabetes mellitus affects approximately 1.1–14.3% of pregnant women^2^,^3^,^4^, and has 35.6% to 69% of recurrence risk^5^,^6^. GDM has adverse effects on the pregnant women and fetus including pre-eclampsia, caesarean section rates, perinatal mortality, birth defects, macrosomia, etc. In longitudinal studies with a duration of at least 5 years, 20% to 65% of women with GDM go on to develop type 2 diabetes (T2DM)^7^. The pathogenesis of GDM is not fully understood, the syndrome has many similarities to T2D that becomes manifest during the course of pregnancy. T2D, also called non-insulin-dependent diabetes (NIDDM), is characterized by hyperglycemia resulting from impairment of insulin secretion and/or defects in insulin action in peripheral tissues. GDM represents a combination of acquired and intrinsic abnormalities of insulin action. The precise mechanisms underlying gestational diabetes are still largely unknown.

MiRNAs are small 19–23 nucleotide RNA molecules that act as negative regulators of gene expression by mediating messenger RNAs degradation or translational arrest. They are potent drivers of differentiation and development in many biological processes. The evidence increasingly shows that miRNA dysregulation has been linked to diabetes in recent years. As is known, miRNAs play roles in type 1 and type 2 diabetes (T1D and T2D), focusing on β-cell biology, insulin resistance and diabetes complications^8^. *MiR-98* expression is up-regulated in kidney in response to diabetes complications in mouse in and down-regulated in muscle in type 2 diabetes with insulin resistance in human^9^,^10^. Also, *miR-98* is found to participate in embryo implantation during early pregnancy^11^. Although *miR-98* is involved in T2DM and early pregnancy in the available literature^9^,^10^,^11^, the relationship between *miR-98* and GDM remains unknown, and roles of *miR-98* in GDM are still unclear.

In this study, we report the relationship between *miR-98* and GDM and investigate the functional roles of *miR-98* in GDM. Additionally, we also test the possible molecular mechanisms in which *miR-98* is implicated.

## Results

### Up-regulation of *miR-98* expression in placental tissues from patients with GDM

**Figure 1:**
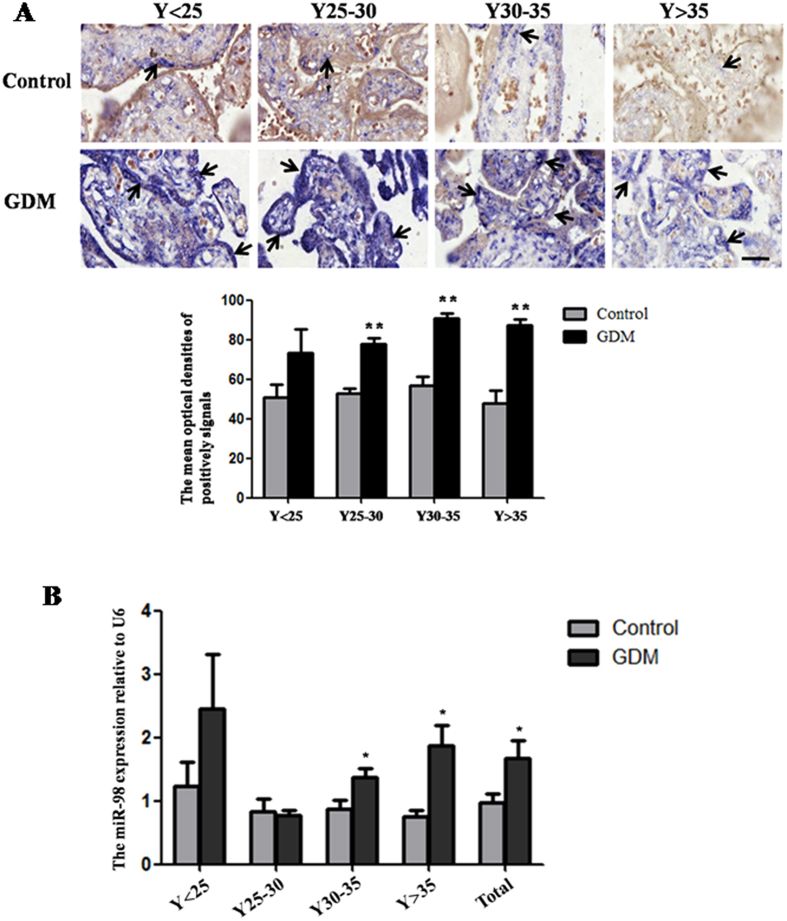
Up-regulation of *miR-98* in the placental tissues from patients with GDM. The expression of *miR-98* in the placental tissues from patients with GDM and normal pregnant women was detected by *in situ* hybridization using DIG-labeled LNA probes specific to *miR-98* (**A**). The stain was developed with BCIP/NBT. Black arrows indicate hybridization signals and the positive signals of *miR-98* are blue. The scale bar indicates a distance of 50 μm. The histogram represents the MODs of positive signals of *miR-98* in placentas. The expression of *miR-98* in the placental tissues was also detected by qRT-PCR (**B**). *U6* serves as an internal reference to normalize the experimental error. *P < 0.05; **P < 0.01.

The distribution of *miR-98* in GDM placental tissues and control tissues was determined by *in situ* hybridization (Fig. 1A). Old age is the risk factor for GDM. Therefore, the GDM tissues were grouped into four groups according to maternal age, including under 25 years old (Y < 25), 25~30 years old (Y25~30), 30~35 years old (Y30~35), over 35 years old (Y > 35). In different age groups, strong signals of *miR-98* were found in GDM placental tissues. While in control group, there was only weak expression of *miR-98* in placenta. The image analysis of optical densities showed that the mean optical densities (MODs) of positive signals were increased in GDM tissues, especially in the groups of Y25~30 (*P* < 0.01), Y30~35 (*P* < 0.01) and Y > 35 (*P* < 0.01), when compared with the corresponding controls.

qRT-PCR was used to further confirm the results of *in situ* hybridization (Fig. 1B). The expression levels of *miR-98* in GDM group were markedly higher than that in control group in Y30~35 (*P* < 0.05) and Y > 35 (*P* < 0.05). The overall expression trend of *miR-98* in all placental tissues of GDM group was consistent with that in Y30~35 and Y > 35 age groups and significantly increased compared with control group (*P* < 0.05). These results imply that *miR-98* is sensitive to occurrence of GDM.

### Demethylation reduces the expression of *miR-98*

**Figure 2:**
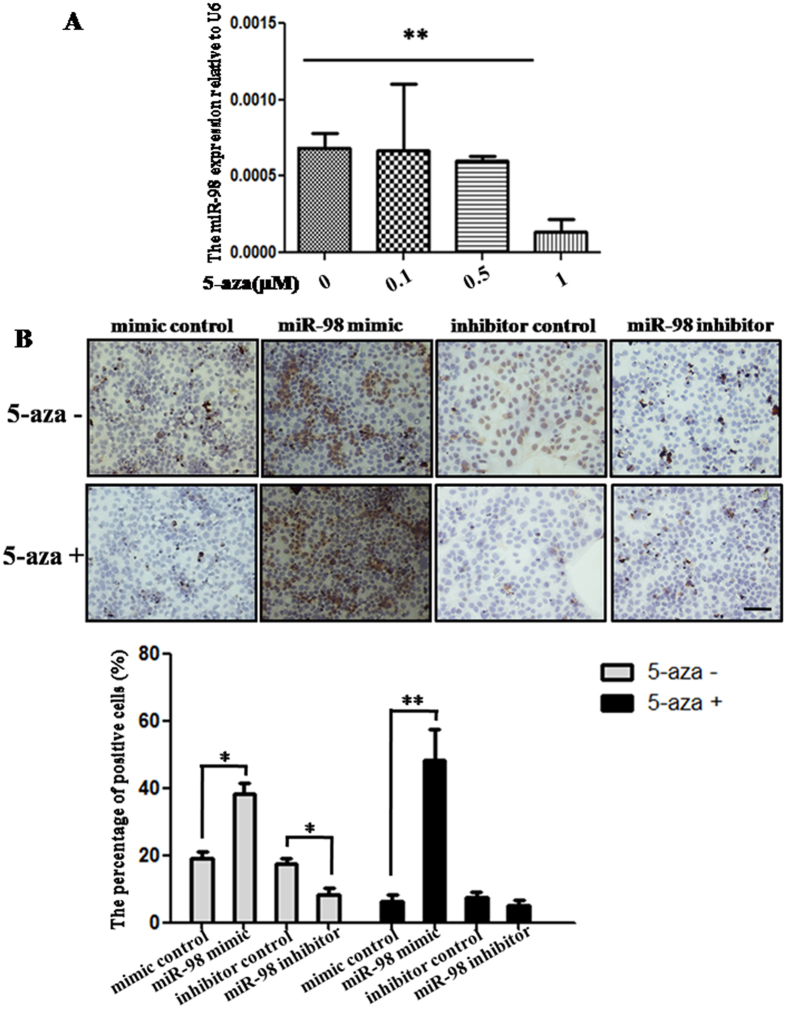
*MiR-98* and DNA methylation level in the placental tissues from patients with GDM. The *miR-98* level was detected in JEG-3 cells treated with 5-aza by qRT-PCR (**A**). *U6* serves as an internal reference to normalize the experimental error. Global DNA methylation level in *miR-98* mimic or inhibitor-treated cells with or without 5-aza was detected by immunohistochemistry and estimated by the content of global 5-meC (**B**). The stain was developed with DAB and cell nuclei were stained with haematoxylin. Brown indicates the positive signals. Scale bar = 50 μm. The histogram represents the ratio of 5-meC positive cells (%). *P < 0.05; **P < 0.01.

In order to analyze the effect of demethylation on the expression of *miR-98*, DNA methylation inhibitor 5-aza was used to treat JEG-3 cells, and then the expression level of *miR-98* was detected by qRT-PCR (Fig. 2A). The results indicated that 0.1, 0.5, 1 μM 5-aza inhibited the expression of *miR-98* in a dose-dependent manner. However, only 1 μM 5-aza significantly inhibited *miR-98* expression (*P* < 0.01). These results suggest that *miR-98* may be related with DNA methylation.

### *MiR-98* enhances the DNA methylation level *in vitro*

To verify the relationship between *miR-98* and DNA methylation, the global DNA methylation level in JEG-3 cells transfected by *miR-98* mimic or inhibitor was estimated by the content of 5-methylcytosine (5-MeC) detected by cells immunohistochemistry (Fig. 2B). The ratio of 5-MeC positive cells was significantly increased in cells transfected by *miR-98* mimic compared with mimic control (*P* < 0.05). *MiR-98* inhibitor markedly reduced the ratio of 5-MeC positive cells (*P* < 0.05). DNA methylation inhibitor 5-aza can reduce the ratio of 5-MeC positive cells. However, transfection of *miR-98* mimic into 5-aza-treated cells significantly enhanced the ratio of 5-MeC positive cells (*P* < 0.01). These results show that *miR-98* can positively regulate the global DNA methylation level.

### *Mecp2* is a direct target of *miR-98*

**Figure 3:**
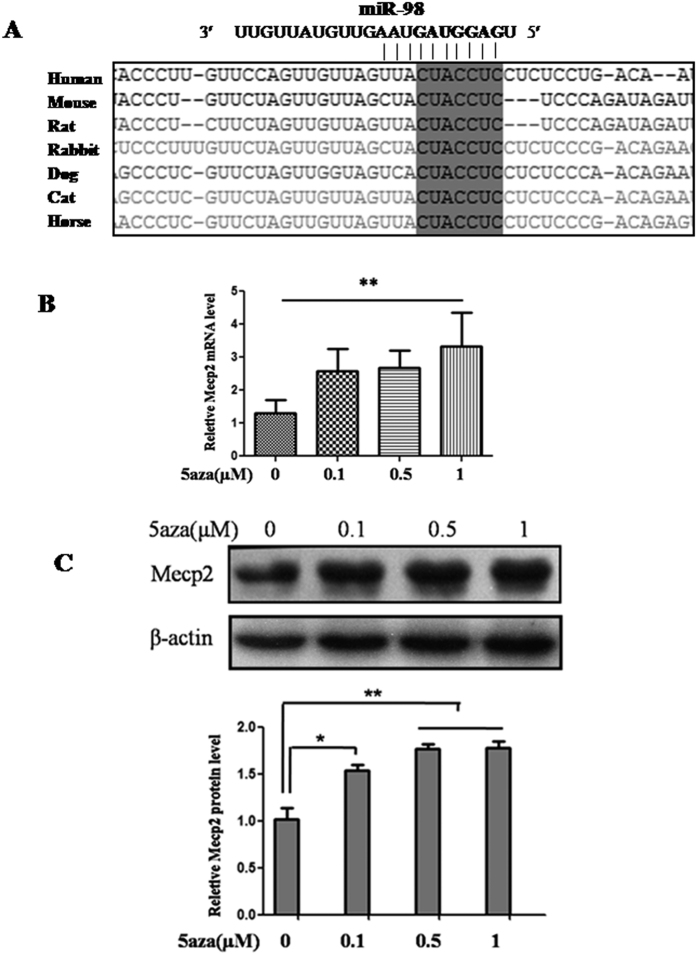
The prediction of the *miR-98* target gene. *MiR-98* binding site in the 3′-UTR region of *Mecp2* was conserved in cross-species (**A**). 5-aza treatment enhanced *Mecp2* mRNA level and protein level detected by qRT-PCR (**B**) and western blot (**C**). *Gapdh* and β-ACTIN serve as an internal reference for qRT-PCR and western blot, respectively. For western blot, the gels had been run under the same experimental conditions. The bands were analyzed using Quantity One analyzing system (Bio-Rad, Hercules, CA, USA). The histogram represents the optical densities of the signals quantified by densitometric analysis and expressed as MECP2 intensity/β-ACTIN intensity to normalize for gel loading and transfer. *P < 0.05; **P < 0.01.

To figure out the possible molecular mechanisms by which *miR-98* may perform in DNA methylation, its target genes were researched. An online search of *miR-98* targets by Targetscan, PicTar and miRanda provided a large number of putative miRNA targets. Among them, we focused on *Mecp2* for the following reasons: (1) Targetscan, PicTar and miRanda prediction showed that there was a *miR-98* responsive element in 3′-UTR of *Mecp2*, which is a highly conserved domain among different species (Fig. 3A). (2) It was reported that *Mecp2* was associated with methylation^12^. (3) In this study, we found that the mRNA and protein level of *Mecp2* was significantly up-regulated when *miR-98* was down-regulated in 5-aza-treated cells (Fig. 3B,C). Thus, an evident inverse relationship is showed between *miR-98* and *Mecp2* expression levels.

**Figure 4:**
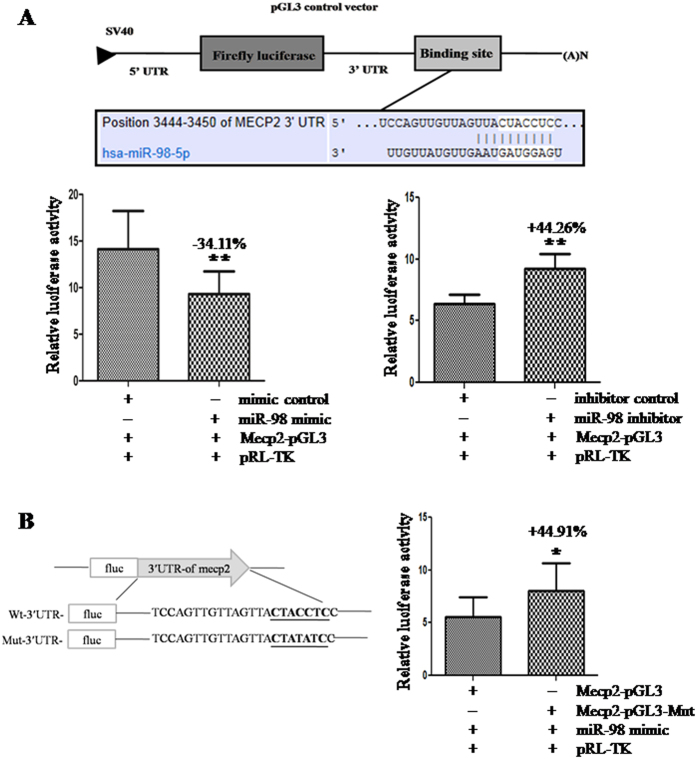
The confirmation of the *miR-98* target. (**A**) The effects of *miR-98* expression dysregulation on *Mecp2* translation. HEK-293T cells were co-transfected with mimic control, *miR-98* mimic, inhibitor control or *miR-98* inhibitor and Mecp2-pGL3 for dual-luciferase assay. (**B**) Mutation analysis of the *miR-98* binding site. Mutating the binding site of *miR-98* in the 3′-UTR of Mecp2 was used as control (Mecp2-pGL3-Mut). PRL-TK containing Renilla luciferase was co-transfected for data normalization.*P < 0.05, **P < 0.01.

**Figure 5:**
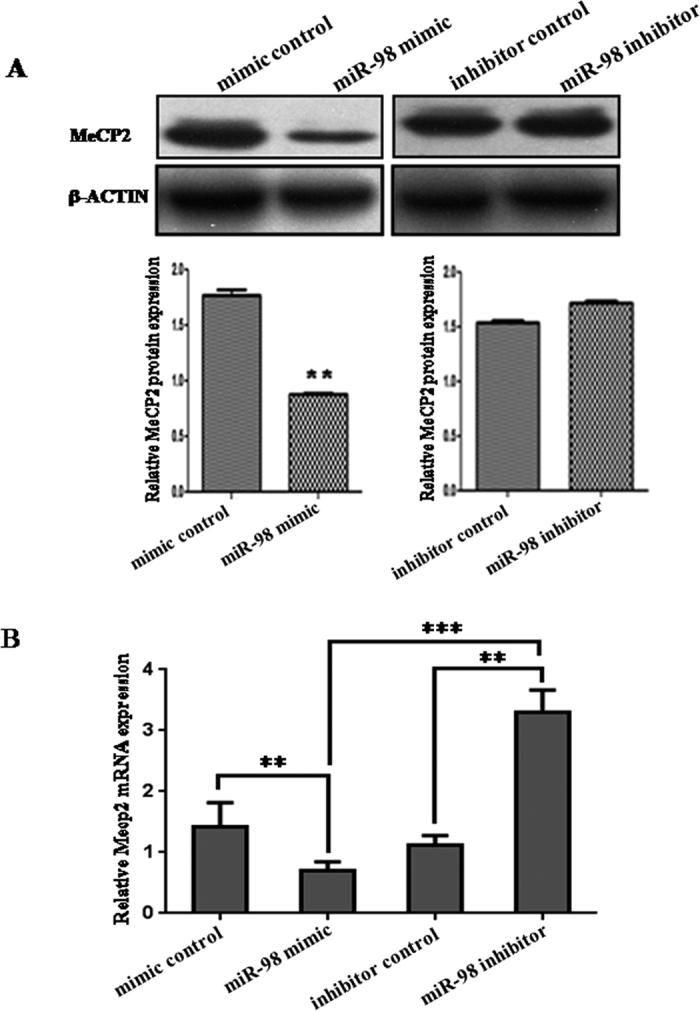
*MiR-98* regulates *Mecp2* expression in *in vitro* cell experiment. (**A**) MECP2 protein level in *miR-98* mimic or inhibitor-treated JEG-3 cells was detected by western blot. The bands were analyzed using the Quantity One analyzing system (Bio-Rad Laboratory inc.). The gels had been run under the same experimental conditions. The expression of β-ACTIN serves as an internal control. (**B**) The mRNA level of *Mecp2* in *miR-98* mimic and inhibitor-treated JEG-3 cells was detected by qRT-PCR. *Gapdh* serves as an internal reference. **P < 0.01. ***P < 0.001.

To validate whether *Mecp2* was the indeed target gene of *miR-98* or not, a human *Mecp2* 3′-UTR fragment containing wild-type was cloned into the downstream of the firefly luciferase reporter gene in the pGL3 control vector (designated as Mecp2-pGL3) for the dual-luciferase assay (Fig. 4A). HEK-293T cells were co-transfected with Mecp2-pGL3 and *miR-98* mimic or inhibitor. Compared with the mimic control, the luciferase activity was significantly suppressed by the *miR-98* mimic, (*P* < 0.01). Furthermore, the luciferase activity was significantly enhanced by the *miR-98* inhibitor compared with inhibitor control (*P* < 0.01; Fig. 5B). These results indicate that *miR-98* affects the binding of *miR-98* and 3′-UTR of *Mecp2*, leading to the change of *Mecp2* translation.

To further confirm the binding site, base mutation of *miR-98* targeting site in 3′-UTR of *Mecp2* (designated as MeCP2-pGL3-Mut) was also conducted. The histogram in Fig. 4B showed that the enzyme activity was significantly reduced in cells co-transfected with *miR-98* mimic and Mecp2-pGL3 compared with Mecp2-pGL3-Mut (*P* < 0.05). These data indicates that *miR-98* may suppress *Mecp2* expression through binding to *miR-98* responsive element in the 3′-UTR of *Mecp2*, and *Mecp2* may be a direct target of *miR-98*.

### *MiR-98* regulates endogenous *Mecp2* expression *in vitro*

Although *Mecp2* was identified as a target gene for *miR-98*, it was unknown whether *miR-98* could regulate endogenous *Mecp2* expression. To verify the endogenous effects of *miR-98* expression dysregulation on *Mecp2*, JEG-3 cells were transfected with *miR-98* mimic or inhibitor. Compared with corresponding control, the level of MECP2 protein was significantly down-regulated by *miR-98* mimic and up-regulated by *miR-98* inhibitor (Fig. 5A). Additionally, the mRNA level of *Mecp2* detected by qRT-PCR was significantly decreased by *miR-98* mimic (*P* < 0.01) and increased by *miR-98* inhibitor (*P* < 0.01). Compared with *miR-98* mimic, *miR-98* inhibitor significantly enhanced *Mecp2* mRNA level (*P* < 0.001; Fig. 5B). These results show that the mRNA and protein levels of endogenous *Mecp2* are regulated by *miR-98*.

### *Mecp2* was down-regulated in GDM placental tissues

**Figure 6:**
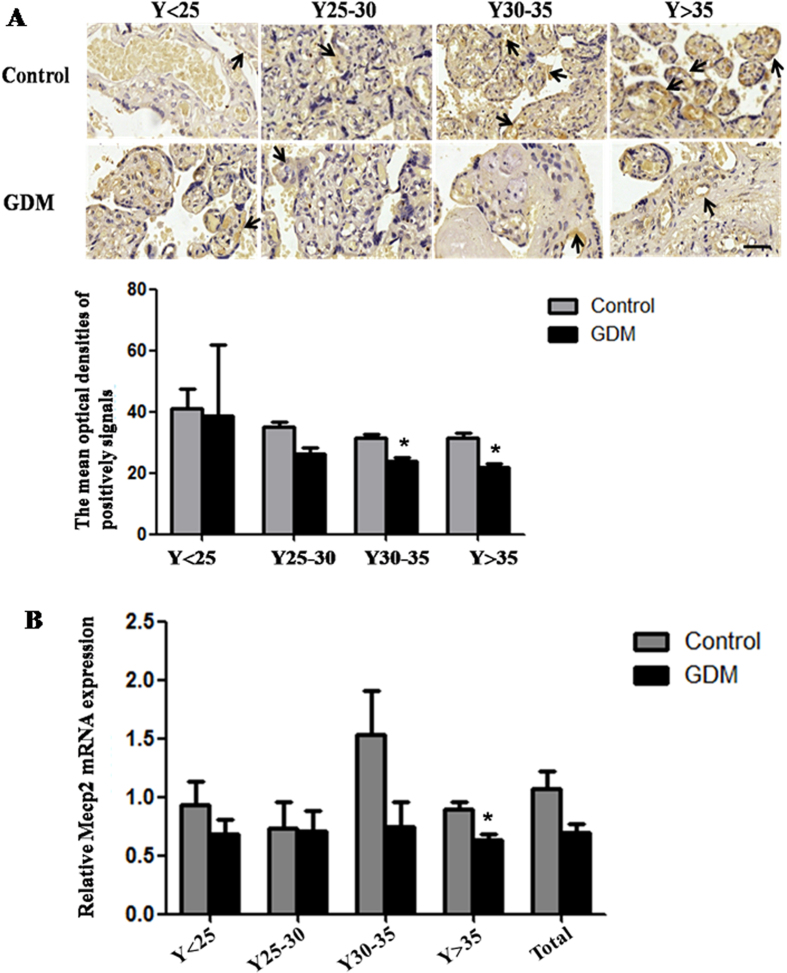
The expression of *Mecp2* in the placental tissues from patients with GDM. (**A**) The protein level of MECP2 in the placental tissues from patients with GDM and normal pregnant women was detected by immunohistochemistry. The stain was developed with DAB and cell nuclei were stained with haematoxylin. Black arrow indicated positive signals and the positive signals of MECP2 are brown. The histogram represents the MODs of positive signals of MECP2 in placentas. Scale bar = 50 μm. (**B**) qRT-PCR was used to detected the mRNA level of *Mecp2* in the placental tissues from patients with GDM and normal pregnant women. *Gapdh* serves as an internal reference. *P < 0.05.

Then it was unclear whether *miR-98* executed its effects by targeting *Mecp2* in GDM *in vivo*? To verify the phenomenon, we tested the MECP2 expression in the placental tissues from patients with GDM by immunohistochemistry using rabbit anti-MECP2 antibody (Fig. 6A). The MODs of MECP2-positive signals were significantly decreased in the placental tissues from GDM patients in Y30–35 and Y > 35 age group compared with the corresponding controls (*P* < 0.05), which was further confirmed by qRT-PCR (Fig. 6B). All these facts show that *Mecp2* expression level is down-regulated in GDM tissues, while *miR-98* expression level is visibly increased in GDM placental tissues, suggesting that *miR-98* may execute its effects by targeting *Mecp2* in GDM *in vivo.*

### *MiR-98* and *Mecp2* regulate the protein expression of DNA methyltransferase

**Figure 7:**
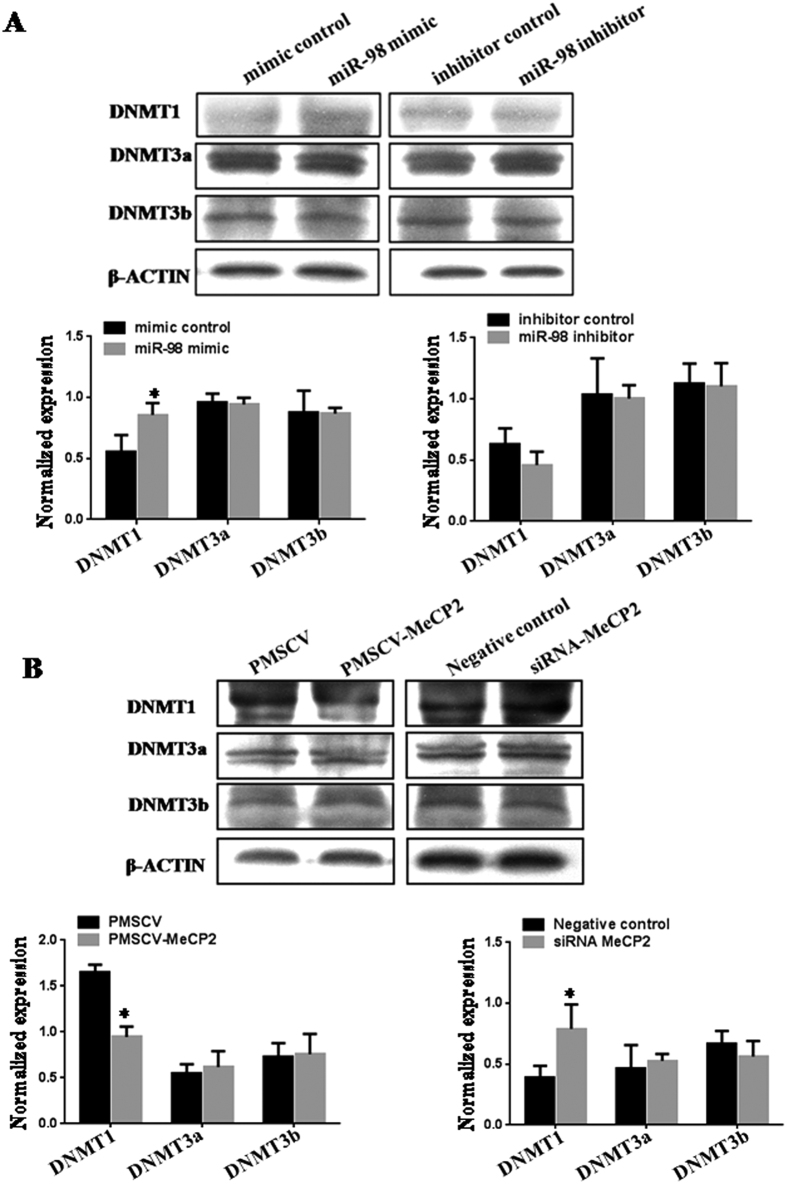
The protein expression of DNA methyltransferase. The effects of *miR-98* (**A**) and *Mecp2* (**B**) on the protein levels of DNA methyltransferase (DNMT1, DNMT3a and DNMT3b) were detected by western blot. The gels had been run under the same experimental conditions. The expression of β-ACTIN serves as an internal control to normalize for gel loading and transfer. The bands were analyzed using Quantity One analyzing system (Bio-Rad, Hercules, CA, USA). The protein level was represented as the relative ratio of the DNMT1, DNMT3a and DNMT3b signals vs β-ACTIN. *P < 0.05.

Because *miR-98* was involved in DNA methylation, we wondered whether *miR-98* and its target gene would affect the protein expression of DNA methyltransferase or not (Fig. 7). *MiR-98* mimic increased DNMT1 protein level (*P* < 0.05) and *Mecp2* expression vector decreased DNMT1 protein level (*P* < 0.05) compared with corresponding control. DNMT1 protein level had a downward tendency in cells treated by *miR-98* inhibitor and a obvious increase by *Mecp2* siRNA (*P* < 0.05). However, *miR-98* and *Mecp2* had no significant effects on the protein levels of DNMT3a and DNMT3B.

### *MiR-98* indirectly regulates the expression of *Trpc3* by targeting *Mecp2*

**Figure 8:**
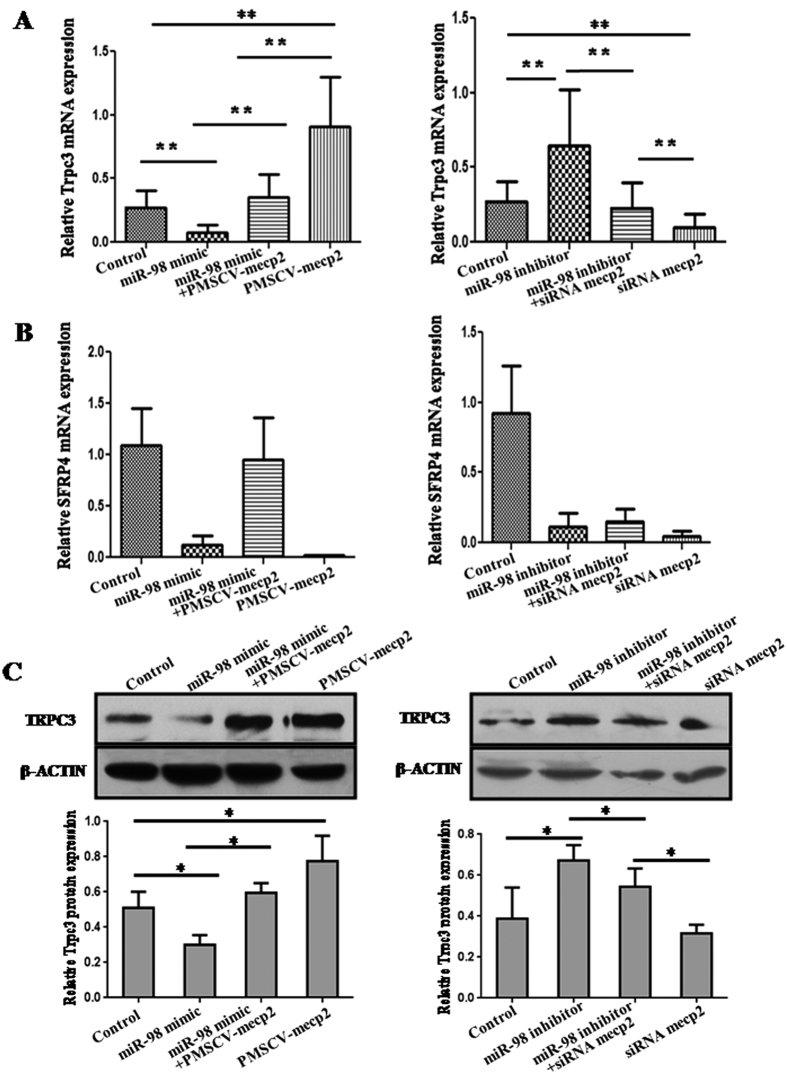
The *miR-98* indirectly regulates *Trpc3* expression by *Mecp2*. qRT-PCR analysis of *Trpc3* (**A**) and *Sfrp4* (**B**) mRNA levels and western blot analysis of TRPC3 protein level (**C**) in *miR-98* mimic or *miR-98* inhibitor-treated cells with *Mecp2* expression vector or *Mecp2* siRNA. *Gapdh* and β-ACTIN serve as an internal reference for qRT-PCR and western blot, respectively. *P < 0.05, **P < 0.01.

Previous studies have identified that canonical transient receptor potential 3 (*Trpc3*) and secreted frizzled-related protein 4 (Sfrp4), which play roles in T2DM, are the target genes of *Mecp2*^13^,^14^. Then it was unknown whether *miR-98* affected the expression of *Mecp2* target genes? Therefore, the expression of *Trpc3* and *Sfrp4* was detected by qRT-PCR (Fig. 8A,B). *MiR-98* mimic significantly reduced the mRNA level of *Trpc3 (P* < 0.01). *Mecp2* expression vector pMSCV-*Mecp2* significantly increased *Trpc3* expression (*P* < 0.01). When cells were co-transfected with *miR-98* mimic and pMSCV-Mecp2, the mRNA level of *Trpc3* were higher than transfection of *miR-98* mimic (*P* < 0.01) and lower than transfection of pMSCV-Mecp2 (*P* < 0.01), implying that *Trpc3* expression suppressed by *miR-98* over-expression was partially rehabilitated by *Mecp2* up-regulation. Additionally, *miR-98* inhibitor significantly promoted the expression of *Trpc3 (P* < 0.01). *Mecp2* siRNA significantly inhibited the expression of *Trpc3 (P* < 0.01). However, the mRNA level of *Trpc3* in cells co-transfected with *miR-98* inhibitor and *Mecp2* siRNA was significantly weakened compared with *miR-98* inhibitor alone (*P* < 0.01) and strengthened compared with *Mecp2* siRNA alone (*P* < 0.01), displaying that *miR-98* low expression-mediated the up-regulation of *Trpc3* was partially attenuated by *Mecp2* knockdown (Fig. 8A). *MiR-98* mimic down-regulated *Sfrp4* mRNA level, while miR-98 inhibitor, *Mecp2* expression vector, *Mecp2* siRNA also inhibited *Sfrp4* expression. So, there was no distinct trend exhibiting the effects of *miR-98* and *Mecp2* on the expression of *Sfrp4* (Fig. 8B).

In order to further confirm that *miR-98* affected the expression of *Trpc3*, the protein level of TRPC3 was detected by western blot (Fig. 8C). Comparable results were obtained by western blot and qRT-PCR. *MiR-98* mimic significantly reduced the protein level of TRPC3 (*P* < 0.05), and pMSCV-*Mecp2* significantly increased TRPC3 protein level (*P* < 0.05). When cells were co-transfected with *miR-98* mimic and pMSCV-Mecp2, the protein level of TRPC3 was higher than transfection of *miR-98* mimic lone (*P* < 0.05) and close to the control. *MiR-98* inhibitor significantly enhanced TRPC3 protein level (*P* < 0.05). TRPC3 protein level had a downward trend in cells treated by *Mecp2* siRNA. However, the protein level of TRPC3 in cells co-transfected with *miR-98* inhibitor and *Mecp2* siRNA was significantly weaker than transfected with *miR-98* inhibitor alone (*P* < 0.05) and stronger than transfected with *Mecp2* siRNA alone (*P* < 0.05). Taken together, these results indicate that *miR-98* executes functions in GDM partially by targeting Mecp2-Trpc3 pathway.

### *Trpc3* expression in GDM placental tissues

**Figure 9:**
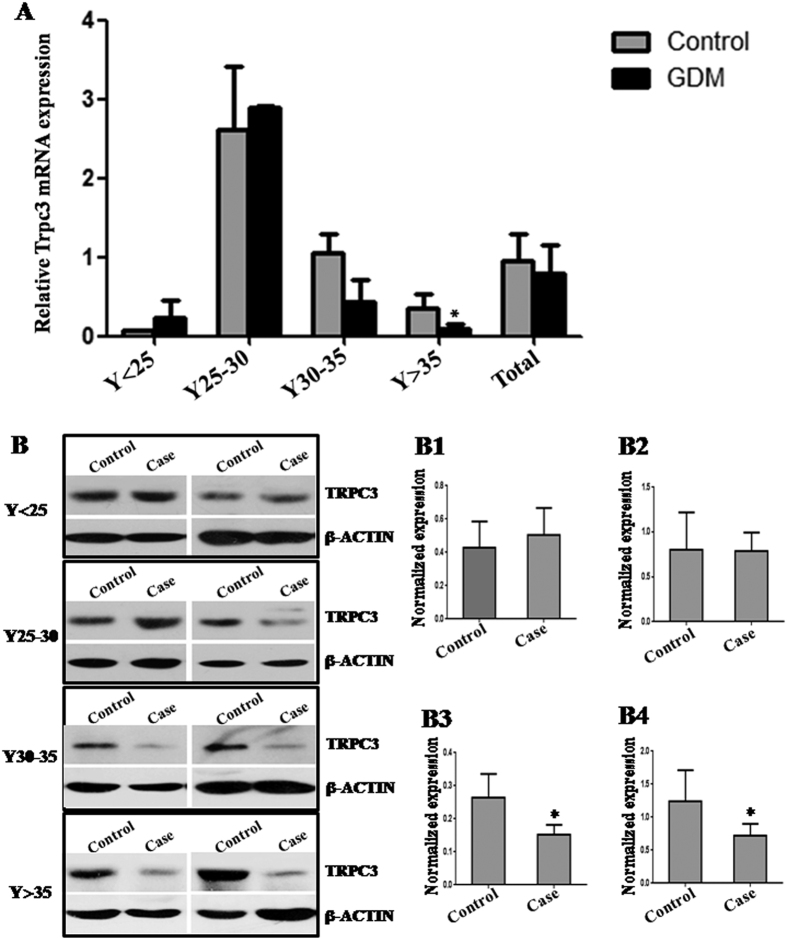
*Trpc3* expression in GDM placental tissues. The *Trpc3* expression in the placental tissues from patients with GDM was detected by qRT-PCR (**A**) and western blot (**B**). The gray histograms of B1–B4 represent the protein level of MECP2 in placental tissues in Y < 25, Y25~30, Y30~35 and Y > 35 age group, respectively. *Gapdh* and β-ACTIN serve as an internal reference for qRT-PCR and western blot, respectively. *P < 0.05.

In order to analyze *Trpc3* expression *in vivo*, we tested the *Trpc3* expression in the placental tissues from patients with GDM by qRT-PCR and western blot (Fig. 9). The *Trpc3* mRNA level was significantly decreased in the placental tissues from GDM patients in Y > 35 age group compared with the normal control (*P* < 0.05; Fig. 9A). The TRPC3 protein level was significantly decreased in the placental tissues from GDM patients in Y30–35 and Y > 35 age group compared with the corresponding controls (*P* < 0.05; Fig. 9B).

## Discussion

In this study, we found that *miR-98* was significantly up-regulated in placental tissues from GDM patients compared with that in normal controls. The trend of *miR-98* in four groups, including Y < 25, Y25~30, Y30~35 and Y > 35, is similar, suggesting that up-regulation of *miR-98* may be associated with the occurrence of GDM.

Interestingly, DNA methylation inhibitor 5-azacytidine could decrease the *miR-98* level in human choriocarcinoma cell line JEG-3. Emerging evidence indicates that GDM has epigenetic effects on genes through DNA methylation, with consequences on fetal growth and development^15^. So we speculate that *miR-98* may be able to regulate DNA mathylation in GDM. *In vitro* cell experiment was used to confirm the relationship between *miR-98* and DNA methylation, and found that over-expression of *miR-98* increased global DNA methylational level, and partially recovered the inhibition of DNA methylation induced by 5-azacytidine. These facts show that enforced *miR-98* expression promotes global DNA methylation level.

It is generally accepted viewpoint that miRNAs function via regulating the expression of their downstream target genes. An online search of *miR-98* targets by Targetscan, PicTar and miRanda found that there was a *miR-98* responsive element in 3′-UTR of *Mecp2*, which was a highly conserved domain among different species. When *miR-98* mimic or inhibitor was co-transfected with the recombinant vector Mecp2-pGL3, luciferase activity was reduced by the *miR-98* mimic and enhanced by the *miR-98* inhibitor, suggesting that *Mecp2* may be the target gene of *miR-98.* Mutation experiment further confirmed that the binding site in the 3′-UTR of *Mecp2* was specific for *miR-98*. Additionally, over-expression of *miR-98* reduced the protein and mRNA level of *Mecp2* and knockdown of *miR-98* enhanced the protein and mRNA level of *Mecp2*. These data further confirm that *miR-98* not only directly targets *Mecp2,* but also regulates the endogenous MECP2 expression.

Previous studies have identified that *Trpc3* and *Sfrp4* are *Mecp2* target gene respectively^13^,^14^. *Trpc3* is involved in vasoconstriction and regulation of blood pressure in metabolic syndrome^16^. *Sfrp4* reduces insulin secretion and may be a potential biomarker for islet dysfunction in T2D^17^. We found that *miR-98* over-expression suppressed *Trpc3* expression, which was partially rehabilitated by up-regulation of *Mecp2. MiR-98* knockdown promoted *Trpc3* expression, which was partially attenuated by depleting *Mecp2* expression. There was no significant effect of *miR-98* on *Sfrp4* expression. A study analyzing insulin-mediated glucose uptake shows that *Trpc3* interacts functionally and physically with GLUT4, and Ca(2+) influx and modulates insulin-mediated glucose uptake^18^. Therefore, *miR-98* may indirectly regulate glucose up-take through targeting Mecp2-Trpc3 pathway.

To our knowledge, this is the first study to examine the relationship between the presence of maternal GDM and *miR-98*. Our findings demonstrate that *miR-98* and its pathways in placental tissues are associated with GDM. *MiR-98* not only directly targets *Mecp2*, but also indirectly regulates the target gene of *Mecp2*. These results imply that enhanced *miR-98* expression may take part in the occurrence of GDM by Mecp2-Trpc3 pathway.

## Materials and Methods

### Patients samples and tissue preparation

Human placentas from 37 weeks~40 weeks (37w~40w) pregnancy were obtained from Maternal and Child Health Hospital, Haidian District, Beijing, from March 2012 to May 2013, including 193 GDM patients and 202 normal pregnant women as control. Basic characteristics of the population were displayed in Table 1. The investigation was conducted according to the principles expressed in the Declaration of Helsinki. The study was approved by Ethics Committee of Research Institute for Family Planning (2011-08) and informed consent was obtained from all participants. Placentas were fixed in 4% paraformaldehyde (PFA) diluted in 0.1 M phosphate-buffered saline (PBS) for immunohistochemical and *in situ* hybridization studies.

### Plasmid construction and transfection

The *Mecp2* 3′-UTR and *Mecp2* 3′-UTR-mutant sequences were amplified by PCR from human genomic DNA using the primers in Table 2. After being double digested with *Spe*I and *Xba*I, the PCR products were cloned into pGL3 control vector (Invitrogen, Carlsbad, CA, USA). The coding region of *Mecp2* sequence was amplified by RT-PCR from total mRNA of human JEG-3 cells using the primers in Table 2. After being double digested with *Bam*HI and *Eco*RI, the PCR product was cloned into PMSCV-puro vector, designated as PMSCV-Mecp2. All the constructs were verified by DNA sequencing. Specific siRNAs for scramble and *Mecp2* were synthesized as a duplex with the following sequence: scramble siRNA, 5′-CUUCUUAGGUGGUUUCUGC-dTdT-3′, *Mecp2* siRNA, 5′-GCAGAAACCACCUAAGAAG- dTdT-3′.

The *miR-98* mimic, mimic control, *miR-98* inhibitor, inhibitor control, scramble siRNA control and *Mecp2* siRNA were synthesized by GenePharma (GenePharma Co., Ltd, Shanghai, China), and transfected into cells by the lipofectamine 2000 (Invitrogen, Carlsbad, CA, USA) according to the manufacture’s instruction.

### *In situ* hybridization

An *in situ* hybridization procedure was performed using the protocol developed in our laboratory. Placentas were arranged into tissue microarray and cut to a thickness of 6 μm. After deparaffinized in xylene, rehydrated in descending ethanol series, refixed in 4% PFA, deproteinized with 0.2 M HCl and digested with 20 μg/ml proteinase K (Tiangen, China), the sections were prehybridized with hybridization buffer (Roche, Mannheim, Germany) at 40 °C for 1 h and then hybridized with digoxigenin (DIG)-labeled LNA-MiR-98 probe (LNA-MiR-98 sequence: 5′–DIG-aAcaaTaCAaCttaCtAcCtCa-3′) overnight at 40 °C. After washed by descending saline-sodium citrate (SSC) and blocked by blocking buffer containing 5% bovine serum albumin (BSA), the sections were incubated with alkaline phosphatase (AP) labelled anti-DIG-antibody (Roche, Mannheim, Germany, 1:250) overnight at 4 °C, and developed with bromochloroindolyl phos-phate/nitro blue tetrazolium (BCIP/NBT; Promega, Madison, WI, USA). BCIP and NBT are the common substrate of alkaline phosphatase. Catalyzed by alkaline phosphatase, BCIP product will be hydrolyzed to produce a strong reactivity, and then the product will react with NBT and form an insoluble blue NBT-formazan. The probe was replaced by DIG-labeled LNA-scrambled probe (LNA-scrambled sequences: 5′-DIG-caTtaAtgTcGgaCaaCtcAat-3′) as negative control. Samples were viewed by Nikon TE 2000-U microscope (NIKON, Tokyo, Japan).

### Immunohistochemistry

Sections of the tissues microarray were deparaffinized in xylene and rehydrated in descending ethanol series. Antigen retrieval was accomplished with 1 N HCl. Slides were incubated with rabbit anti-MECP2 polyclonal antibody (GeneTex, USA, 1:500) and mouse anti-5 methylcytosine monoclonal antibody (Santa Cruz, USA, 1:100), respectively, then incubated with HRP-conjugated goat anti-rabbit IgG and goat anti-mouse IgG (Jackson Immunoresearch Laboratories, West Grove, PA, USA). The antibody stains were developed by addition of diaminobenzidine (DAB; Sigma-Aldrich, St. Louis, MO, USA) and cell nuclei were stained with haematoxylin (Sigma-Aldrich, St. Louis, MO, USA). The sections were incubated with normal goat serum as negative control. Samples were viewed using under Nikon TE 2000-U microscope (NIKON, Tokyo, Japan). The MODs of positive signals were determined by NIS-Elements BR Image processing and analysis system (NIKON, Japan).

### Quantitative reverse-transcriptase polymerase chain reaction (qRT-PCR)

Total RNAs from tissues and cells were extracted using Trizol (Invitrogen, Carlsbad, CA, USA) accordint to the manufacturer’s protocols. 1 μg of total RNA was subjected to reverse transcription of mRNAs using dT18 as primer and reverse transcription kit (TakaRa Biotechnology (Dalian) Co., Ltd. Dalian, Liaoning, China) to generate total cDNA. Then the mRNA quantitative PCR was carried using primers in Table 2 and FastStart Universal SYBR Green Master (Invitrogen, Carlsbad, CA, USA) using StepOne^TM^ Real-Time PCR System (Applied Biosystems, Foster City, CA, USA). *Gapdh* was used for normalization. The reverse transcription and quantitative PCR of miRNA were carried using special probes for *miR-98* and *U6* (Applied Biosystems, Foster City, CA, USA) with TaqMan MicroRNA Reverse Transcription Kit and Universal PCR Master Mix (Applied Biosystems, Foster City, CA, USA). The quantification was normalized to an endogenous control U6. Each sample in each group was detected in triplicate. The experiment was repeated at least three times.

### Western blot analysis

Extracted protein was boiled in SDS/β-mercaptoethanol sample buffer, and 50 μg protein was run on 5–12% gradient polyacrylamide gel and transferred to PVDF membranes (Amersham, St Albans, Herts, UK). Membranes were incubated with rabbit anti-MECP2 polyclonal antibody (GeneTex, USA, 1:1000), mouse anti-β-ACTIN monoclonal antibody (Abcam, Cambridge, MA USA, 1:1000), rabbit anti-DNMT1, DNMT3a and DNMT3b polyclonal antibody (1:1000, SantCruz Biotechnology Inc., CA, USA) overnight at 4 °C. After that, the membranes were incubated with horseradish peroxidase (HRP)-conjugated goat anti-rabbit IgG or goat anti-mouse IgG (Jackson Immunoresearch Laboratories, Inc., West Grove, PA, USA, 1:10, 000) at room temperature for 1 h. ECL detection reagents (Millipore, Billerica, MA, USA) were added on the membranes for 1 min and were immediately exposed to X-ray film (Kodak, USA). The β-actin signal was used as a loading control. The experiment has been repeated at least three times. The bands were analyzed using Quantity One analyzing system (Bio-Rad, Hercules, CA, USA). The protein level was represented as the relative ratio of the MECP2, DNMT1, DNMT3a and DNMT3b signals vs the housekeeping gene (β-ACTIN).

### Dual-luciferase activity assay

To generate 3′-UTR luciferase reporter, partial sequence of the 3′-UTR from *Mecp2* were cloned into the downstream of the firefly luciferase gene in pGL3-Control Vector (Promega, Madison, WI, USA) using primers in Table 2. Mutating *miR-98* target site in the 3′-UTR of *Mecp2* was used as control. PRL-TK containing Renilla luciferase was co-transfected for data normalization. For luciferase reporter assay, HEK-293T cells were seeded in 48-well plates and allowed to attach overnight, then transfected using lipofectamine 2000 (Invitrogen, Carlsbad, CA, USA). Two days later, cells were harvested and assayed with the dual-luciferase assay (Promega, Madison, WI, USA). Each treatment was performed in triplicate in three independent experiments. The results were expressed as relative luciferase activity (Firefly LUC/Renilla LUC).

### Statistical analyses

All statistical analysis were performed using the SPSS 13.0 statistical software package. Data are presented as mean ± SEM from at least three independent experiments. Multiple group comparisons were performed using one-way analysis of variance (ANOVA), and two group comparisons were performed using T-test. Differences were considered statistically significant at *P* < 0.05.

## Additional Information

**How to cite this article**: Cao, J.-L. *et al.* Up-regulation of *miR-98* and unravelling regulatory mechanisms in gestational diabetes mellitus. *Sci. Rep.* **6**, 32268; doi: 10.1038/srep32268 (2016).

## Figures and Tables

**Table 1:** Basic characteristics of study population according to GDM.

	Control, n = 202			GDM, n = 193		
	Mean ± SD	95% Confidence Interval for Mean		Mean ± SD	95% Confidence Interval for Mean	
		Lower Bound	Upper Bound		Lower Bound	Upper Bound
Age (years)	29.68 ± 3.66	29.17	30.19	30.93 ± 3.45	30.45	31.42
Height (cm)	161.69 ± 4.95	161.01	162.38	161.59 ± 4.81	160.91	162.27
Body Weight (Kg)	55.32 ± 8.87	54.09	56.55	59.82 ± 8.87**	58.57	61.07
BMI (Kg/m2)	21.06 ± 2.90	20.66	21.46	22.97 ± 3.09**	22.53	23.4
Basic systolic (mmHg)	114.99 ± 10.82	113.48	116.49	117.23 ± 11.34*	115.63	118.83
Foundation diastolic (mmHg)	70.96 ± 9.04	69.71	72.21	72.90 ± 8.93	71.64	74.16
Glu first detected	4.52 ± 0.38	4.47	4.58	4.79 ± 0.58*	4.7	4.87
Fasting plasma glucose	4.53 ± 0.37	4.48	4.58	5.40 ± 0.72**	5.29	5.5
OGTT-1 h	7.54 ± 1.24	7.37	7.72	10.99 ± 1.44**	10.78	11.2
OGTT-2 h	6.26 ± 0.97	6.12	6.39	9.07 ± 1.31**	8.88	9.26
Pregnancy Days	274.41 ± 8.03	273.29	275.52	272.22 ± 6.39	271.32	273.12
Newborn baby’s Weight	3391.73 ± 433.08	3331.65	3451.82	3435.54 ± 475.04	3368.1	3502.99

*P* < 0.05; *P* < 0.01.

**Table 2:** Primer sequences.

Gene Name	Primer Sequence	Accession Number	Size and Location	Application
MeCP2 3′-UTR	Forward/SpeI:5′-GACTAGTCCCACCCTTGTTCCAGTTGT-3′	NM_004992.3	365 bp (5105–5469)	PCR
	Reverse/XbaI:5′-GCTCTAGATGCCACCCAACAGAAGATT-3′			
MeCP2 3′-UTR (mutation)	Forward:5′-GTTCCAGTTGTTAGTTACTATATCCTCTCCTGACAATACT-3′	NM_004992.3	365 bp (5105–5469)	PCR
	Reverse:5′-TATAGTAACTAACAACTGGAACAAGGGTGGG-3′			
MeCP2 (coding region)	Forward/kozak-BamHI: 5′-CGCGGATCGCCACCATGGTAGCTGGGATGTTAGGGCT-3′	NM_004992.3	1461 bp (227–1687)	RT-PCR
	Reverse/EcoRI: 5′-CCGGAATTCTCAGCTAACTCTCTCGGTCACGG-3′			
MeCP2	Forward: 5′-TGCAAAGAGGAGAAGATGCCCAGA-3′	NM_004992.3	169 bp (1463–1631)	qRT-PCR
	Reverse: 5′-GCCTTGGCATGGAGGATGAAACAA-3′			
Trpc3	Forward: 5′-CATTCCTGGCCATTGGCTACT-3′	NM_001130698.1	117 bp (1375–1491 bp)	qRT-PCR
	Reverse: 5′-GCAGACCCAGGAAGATGATGAA-3′			
Gapdh	Forward: 5′-TGGTATCGTGGAAGGACTCA-3′	NM_002046.4	129 bp (678–806)	qRT-PCR
	Reverse: 5′-GTAGAGGCAGGGATGATGTTC-3′			
